# Development of an integrated digital health intervention to promote engagement in and adherence to medication for opioid use disorder

**DOI:** 10.1186/s13722-020-00189-4

**Published:** 2020-04-29

**Authors:** Kirsten J. Langdon, Susan Ramsey, Caroline Scherzer, Kate Carey, Megan L. Ranney, Josiah Rich

**Affiliations:** 1 Department of Psychiatry, Rhode Island Hospital, 146 West River Street, Suite 11A, Providence, RI 02904 USA; 2 Department of Psychiatry and Human Behavior, Alpert Medical School of Brown University, Providence, USA; 3 Department of Medicine, Alpert Medical School of Brown University, Providence, USA; 4 Division of General Internal Medicine, Department of Medicine, Rhode Island Hospital, Providence, USA; 5 Department of Emergency Medicine, Alpert Medical School Brown University, Providence, USA; 6 Department of Behavioral and Social Sciences, Brown University School of Public Health, Providence, USA; 7 Brown University School of Public Health, Center for Alcohol and Addiction Studies, Providence, USA; 8 Emergency Digital Health Innovation Program, Brown University, Providence, USA; 9 Department of Epidemiology, Brown University School of Public Health, Providence, USA

**Keywords:** Opioid use disorder, Buprenorphine, Medications for opioid use disorder, Distress tolerance, Motivational enhancement, Mobile treatment

## Abstract

**Background:**

Buprenorphine-naloxone is an evidence-based treatment for Opioid Use Disorder. However, despite its efficacy, nearly half of participants are unsuccessful in achieving stabilization (i.e., period of time following medication induction in which medication dose is adjusted to be effective in reducing cravings/withdrawal, minimize potential side effects, and eliminate illicit substance use). This paper presents the study design and protocol for a digital health intervention designed to promote engagement in and adherence to buprenorphine treatment, offered through an outpatient addiction treatment center, through motivational enhancement and distress tolerance skills training. Personalized feedback interventions represent a promising method to effectively motivate engagement in and adherence to buprenorphine treatment. These interventions are generally brief, individually tailored, and have the potential to be delivered via mobile platforms. Distress tolerance, a transdiagnostic vulnerability factor, has been implicated in the development and maintenance of substance use. Targeting distress tolerance may improve substance use treatment outcomes by promoting the ability to persist in goal-directed activity even when experiencing physical or emotional distress.

**Methods:**

The study aims are to: (1) develop and refine an interactive computer- and text message-delivered personalized feedback intervention that incorporates distress tolerance skills training for persons who have elected to initiate outpatient buprenorphine treatment (iCOPE); (2) examine the feasibility, acceptability, and preliminary efficacy of iCOPE for increasing abstinence, adherence, and retention in treatment compared to a treatment as usual comparison condition; and, (3) examine potential mechanisms that may underlie the efficacy of iCOPE in improving outcomes, including motivation, distress tolerance, self-regulation, and negative affect.

**Discussion:**

Results of this study will be used to determine whether to proceed with further testing through a large-scale trial. This work has the potential to improve treatment outcomes by reducing illicit opioid use, increasing adherence/retention, and preventing future overdose and other complications of illicit opioid use.

*Trial Registration* NCT03842384

## Background

The opioid overdose epidemic continues to surge across the country, resulting in a record number of fatal overdoses in 2017 [1]. With more than 2 million individuals meeting criteria for opioid use disorder (OUD) [2], there is need to expand access to evidence-based treatments. Medications for opioid use disorder (MOUD) is the current gold standard treatment for this population [3]. Of the FDA-approved MOUD, buprenorphine-naloxone (buprenorphine), a long-acting partial opioid agonist, has grown in popularity because of its more flexible administration through office-based programs [4]. Buprenorphine works by reducing cravings, alleviating withdrawal symptoms, and blocking the euphoric effects of opioids [5]. Meta-analyses highlight the effectiveness of buprenorphine in reducing adverse outcomes [6]. However, a significant proportion of patients return to illicit opioid use and/or discontinue treatment before achieving stabilization (i.e., period of time following medication induction in which medication dose is adjusted to be effective in reducing cravings/withdrawal, minimize potential side effects, and eliminate illicit substance use) [5, 7, 8]. This is notable as early lapses are related to poor treatment prognosis and retention [8, 50].

A negative reinforcement model of substance use posits that individuals may use opioids to mitigate aversive internal states [8, 9]. Consistent with this perspective, negative affect has been cited as a primary precipitant of early lapse among opioid users [10]. Distress tolerance (DT), defined as the perceived or actual ability to handle uncomfortable physical or emotional states, is a transdiagnostic vulnerability factor implicated in the development and maintenance of affective symptoms/disorders and substance use [11, 12]. DT involves the ability to withstand negative reinforcement opportunities by exerting control over behavioral responding that would otherwise provide immediate relief from distress [13]. DT is inversely related to a range of drug use outcomes, including frequency and severity of use [14, 15], premature termination of treatment [16], and early lapse/relapse [17, 18]. Preliminary work suggests that addressing DT during substance use treatment may improve outcomes by promoting the ability to persist in goal directed activity even when experiencing distress and discomfort [19].

Personalized feedback interventions (PFI), such as decisional balance feedback, represent a promising method to effectively motivate engagement in and adherence to buprenorphine treatment, particularly among individuals characterized by low DT. Decisional balance feedback involves an evaluation of the advantages/disadvantages of engaging in a certain behavior (e.g., opioid use), compared to the advantages/disadvantages of an alternative behavior (e.g., abstinence), and also offers strategies for changing problematic behavior (e.g., DT skills training) [20, 21]. Although PFIs have shown promise for reducing substance use across a variety of adult populations [21, 22], no integrated protocols exist to enhance motivation and facilitate DT skills training among individuals with OUD.

Given that negative internal states may occur in participants’ natural environments in response to drug cues, stressors, or the context of withdrawal, this population may benefit from a motivational and DT-based intervention that can be delivered on a mobile platform. In the United States almost 95% of adults own a mobile phone [23]. Text message interventions have demonstrated efficacy for health promotion and behavior change, with personalized messages showing the greatest benefit [24]. These platforms also offer the advantage of being delivered outside of structured treatment sessions and allow for the content, timing, and frequency of messages to be individually tailored to times when certain skills and motivational reminders are most salient [25].

This paper describes the study design and procedures for a behavioral treatment development trial that examines the feasibility, acceptability, and preliminary efficacy of a digital health intervention (iCOPE) designed to promote engagement in and adherence to buprenorphine through motivational enhancement and DT skills training among individuals initiating outpatient addiction treatment.

## Methods

### Study design overview

The current study meets the objectives of Stage I of the NIDA Behavioral Therapies Development Program, which provides a conceptual framework grounded in basic science, for intervention development [26]. Stage I includes two phases—Stage 1A and Stage 1B. Stage 1A involves the creation of a new intervention, while Stage 1B establishes feasibility through pilot testing [26]. In Phase 1 (Stage 1A), our team will develop and refine, through formative evaluation, an interactive computer- and text message-delivered PFI that incorporates DT skills training for persons who have elected to initiate outpatient buprenorphine (iCOPE). Specifically, this intervention is designed to promote medication induction and stabilization following standard clinic intake procedures. All participants will receive standard outpatient buprenorphine treatment, including medication visits, counseling, case management, and peer recovery (treatment as usual; TAU). In Phase 2 (Stage 1B), we will conduct a pilot randomized controlled trial. Eighty patients with a history of OUD, actively seeking outpatient buprenorphine, will be randomly assigned to iCOPE or TAU, using a 3:1 randomization ratio. We propose a 3:1 randomization ratio to maximize the information gained about the iCOPE intervention while including a comparison condition. All participants will complete assessments at treatment initiation as well as 1-, 4-, 8- and 12-weeks post-initiation. These data will be used to inform the development of a large-scale, fully-powered trial. At the time of this report, recruitment for Stage 1A is ongoing.

### Stage 1A

Qualitative, in-depth, individual interviews are currently being conducted with patients actively engaged in buprenorphine treatment (target *N* = 24). The sample is balanced by gender, primary type of opioid use (prescription pills; heroin), and phase of recovery [early (≤ 8 weeks of treatment) vs. late (> 8 weeks of treatment)]. Interviews will continue until saturation is achieved. Following informed consent, demographic and clinical data are obtained. Interviews are conducted by the Principal Investigator (KJL) or a trained Research Assistant, and follow a semi-structured interview protocol that stems from primary research questions and project goals. Specific questions focus on participants’ use of computers, mobile phones, and other technologies; prior engagement in MOUD; barriers/facilitators to engaging in MOUD; reactions to and perceived usefulness of proposed intervention; and preferences, benefits, and likelihood of engaging in digital health interventions. All interviews are conducted in a private office to ensure confidentiality and are digitally recorded. Each interview takes approximately 60–90 min to complete; participants receive a $30 gift card for their time/effort. Interviews are transcribed by a professional agency, and the written transcripts are later reviewed to resolve discrepancies.

Qualitative analyses will be conducted to inform intervention development and refinement. Both thematic (deductive) and data driven (inductive) codes will be utilized. Deductive codes will be drawn from the topics in questions used to facilitate the interviews; inductive codes will capture additional concepts that emerge from the participants. Early interviews will be coded by three team members, until stability of the coding structure is reached. All interviews thereafter will be independently coded by two team members using the coding scheme, then compared to ensure agreement. Agreed upon codes will be entered into NVivo. Throughout the process, a framework matrix will be created. This data reduction tool, a matrix of cases and themes based on interview debriefs and individual interview codes, will be used to track emergent ideas and concepts that impact intervention design and future interviews [27–29]. After every few interviews, research team members will examine the framework matrix, identify reoccurring major themes, make changes to intervention content as appropriate, then test the edits in subsequent interviews. This method will allow for quick, iterative turnaround of participant feedback to intervention edits and modifications of interview questions.

We will further refine the intervention by recruiting an additional 16 participants for open pilot testing. These participants will be in the initial 8 weeks of treatment and will be balanced by gender and primary type of opioid use. At the end of the iCOPE intervention, semi-structured interviews, in combination with measures of protocol adherence, will be used to evaluate and then improve upon the intervention’s appropriateness and comprehensibility. During the interviews, participants will be queried about their comfort with technology (computer and text message); ease of using the technology formats; level of understanding of intervention content; perceived usefulness of material; satisfaction with format and content; likelihood of recommending the intervention; likelihood of continued use; and suggestions for improvement. To guide refinements, we will collect data on the feasibility and acceptability of the intervention. Feasibility will be determined by assessing study recruitment and refusal rates, intervention completion, follow-up rates, and rates of study attrition. Acceptability will be determined by the (a) System Usability Scale, a participant-completed, reliable and valid metric for measuring usability and acceptability of technologies; [30–32] and (b) Relative Subjective Count, the quotient of the participant’s estimate of the number of times the system delivers a text, divided by the number of texts actually delivered [33]. All proposed changes will be integrated into the final version of iCOPE for testing in Phase 2.

### Stage 1B

Stage 1B will employ a randomized controlled trial design to pilot test iCOPE relative to the TAU control condition (see description of treatment conditions below). Adult patients (*N *= 80) meeting eligibility criteria will be randomly assigned to either the iCOPE or TAU conditions.

Following the initial evaluation, in which patients are admitted to buprenorphine treatment, staff will refer potentially eligible patients to study personnel. Interested participants will meet with study personnel in a private setting at the clinic. Informed consent will be obtained, and final eligibility will be determined. Eligible participants will complete a baseline assessment, including two brief behavioral measures of DT (Mirror Tracing and Breath Holding; see Assessments). Participants will then be randomly assigned to iCOPE or TAU. Participants in the iCOPE condition will complete the brief computerized intervention. They will also be informed about the text message element of the intervention and instructed in these procedures. We will recruit 80 participants who will be assigned to condition at a 3:1 ratio, with 60 participants assigned to iCOPE and 20 participants assigned to TAU. Participants will be scheduled to return to the clinic for follow-up appointments consisting of an online survey, behavioral DT measures, and urine collection. Follow-ups will occur at 1-, 4-, 8- and 12-weeks post-treatment initiation.

### Participants

Participants will include 80 males and females between the ages of 18 and 75 initiating OUD treatment, specifically the use of buprenorphine. For inclusion, participants must meet current Diagnostic and Statistical Manual of Mental Disorders, 5th Edition (DSM-5), criteria for OUD and have access to a cell phone with text message capability. To reduce the risk of adverse events and potential confounds, we will employ the following exclusion criteria: (1) active suicidality and/or psychosis that would interfere with the ability to participate in the intervention, (2) not fluent in English as translation is beyond the current study resources, and (3) limited mental capacity or inability to provide informed written consent.

### Study setting

The Lifespan Recovery Center at Rhode Island Hospital will serve as the proposed recruitment site. The Lifespan Recovery Center is an outpatient addiction treatment program that offers buprenorphine and ancillary services (e.g., counseling, case management, peer recovery). The clinical team includes: four physicians, four therapists, a case manager, and a peer recovery specialist. On average, there are approximately 20 new patient admissions per month. As part of routine clinical care at the Lifespan Recovery Center, patients typically attend an intake evaluation and medication induction appointment, followed by weekly follow-up visits for the initial 8 weeks of treatment (stabilization). Once stabilized, patients continue to attend monthly follow-up appointments for the duration of treatment.

### Compensation and retention

In an effort to bolster study retention, we have aligned study follow-up assessments with routine clinic visits. Additionally, follow-up reminders will occur via emails, telephone calls, and text messages. Participants will earn a $25 gift card for completing the initial assessment and a $20 gift card for completing each follow-up assessment. Participants completing all required follow-ups will earn an additional $25 gift card. Participants have the potential to earn up to $130 for completing all study assessments.

## Intervention conditions

### iCOPE

**Fig. 1:**
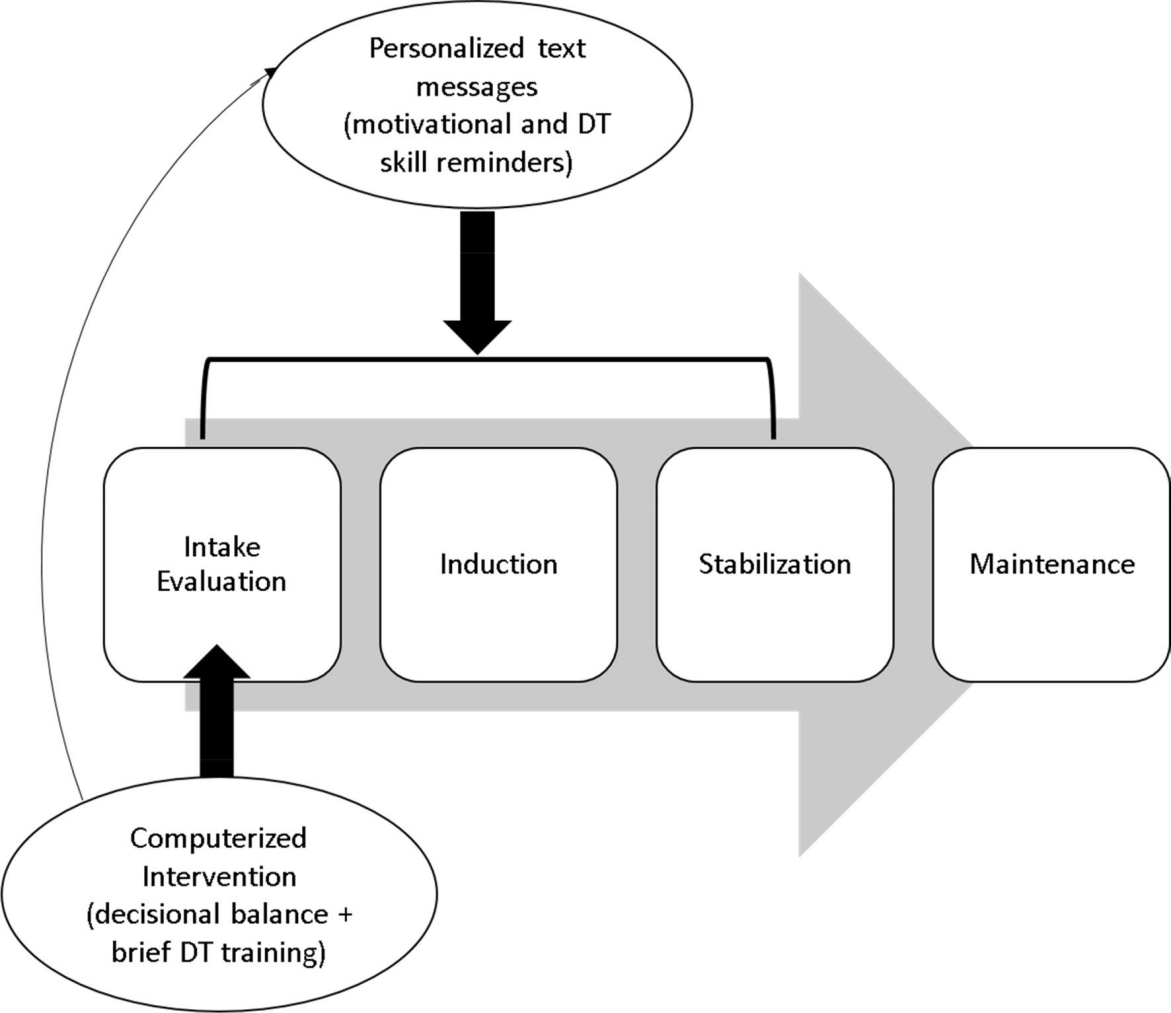
Overview of Proposed iCOPE Intervention

iCOPE will involve two delivery modes: computer and text message (see Fig. 1). The proposed intervention will initially target motivational processes in combination with introductory strategies for managing physical and emotional distress through a single, brief, computer-delivered, session followed by 8 weeks of theoretically-informed text messages intended to enhance motivation and promote DT (see Table 1).

**Table 1:** Description of iCOPE treatment content

Intervention type	Computer intervention components	Text message intervention components
PFI	Evaluate advantages and disadvantages of behavior change	Reminders of the advantages of behavior change elicited during initial intervention
	Elicit recognition of problem and motivating factors for change	Reminders of personal motivators
	Assist with setting personal change goals for opioid use and treatment	Reminders of personal goals
DT	Acceptance: education on meaning of acceptance; strategies to promote acceptance of reality	Acceptance: encourage acceptance-based strategies to use in moments of distress
	Self-soothing: education on using five senses to comfort self during times of distress	Self-soothing: suggestions of ways to comfort self during times of distress
	Distraction: education on using distraction skills to change emotional response to distressing stimuli	Distraction: reminders of distraction strategies to change emotional responding
	Improving the moment: education on methods to replace immediate negative events/states with more positive ones	Improving the moment: encourage methods to replace negative with positive states

*PF* personalized feedback, *DT* distress tolerance

The initial intervention phase will be delivered via computer following routine intake procedures at the recruitment site. We elected to have participants complete the computer-based intervention on-site, instead of allowing participants to complete independently on their own device, to optimize fidelity of the intervention given that it is a Stage 1 trial. We will evaluate participant preference for computer delivery in the open pilot trial to determine if expanding the intervention to home devices is warranted in the future. iCOPE will be presented in a way that encourages participants to be mindful of their behaviors in a non-confrontational manner. The intervention has two aims: (a) engage patients in a decisional balance exercise designed to evaluate the perceived advantages/disadvantages of making a behavior change (abstinence from opioids, adhering to buprenorphine) to enhance motivation and (b) provide concrete strategies to better tolerate emotional and physical discomfort to persist with identified behavioral goals while initiating treatment. First, participants will be guided through a decisional balance exercise and queried about perceived barriers to treatment. Next, this information will be summarized to deliver feedback about personal motivators and recommended DT coping skills.

The second intervention phase will be delivered via text message and will focus on (a) promoting motivation for abstinence over and above the reinforcing effects of opioids and (b) emphasizing adaptive strategies for tolerating physical and psychological discomfort. To do this, text messages will be strategically delivered during the initial 8 weeks of stabilization which are considered a “high-risk” period (see Fig. 1). The text messages will be personalized in nature and offer skills training (reminders of previously learned DT skills) and motivational messages (content directly derived from decisional balance exercise; see Table 1). The frequency and timing of text messages will be determined through prior formative work; however, we anticipate sending at least two automated text messages each day. A variety of messages will be delivered throughout the eight-week period and will create (a) a natural progression in skill-building, (b) continued motivation for engagement, and (c) responsiveness to participants’ needs through personalization. We will create a variety of text messages that provide concrete DT skills as well as personalized messages reminding participants of their identified goals and personal motivators (e.g., health, family, finances). Further, we anticipate providing participant-driven text messages to provide tailored support in the moments needed. For example, if a participant texts “CRAVING” or “STRESS” to the automated program, indicating the need for additional support on that topic, the participant would receive a relevant skills-based message. Messages will be delivered to participants’ personal cell phones using an automated computerized system based upon pre-specified delivery algorithms [34, 35].

### Treatment as usual (TAU)

The TAU condition will consist of comprehensive outpatient buprenorphine treatment, including medication management, counseling, case management, and peer recovery. TAU was selected as the comparison condition as it represents a robust and evidence-based approach to the treatment of OUD. The goal of this phase of treatment development is to evaluate the initial feasibility, acceptability and preliminary efficacy of the proposed intervention through a small pilot trial. Decisional frameworks used to select an appropriate control condition for randomized controlled trials for behavioral interventions suggest that at earlier stages of treatment development it may be favorable to select a TAU condition. Inclusion of a rigid control condition (e.g., health education condition) could result in the underestimation of the effect size and yield null findings in a trial with insufficient statistical power to detect effects [36]. TAU is currently the gold standard of care for treating this population, and therefore, an adequate control condition for this stage of development and evaluation.

## Assessments

### Diagnostic and screening assessment

During routine clinic procedures, patients are evaluated for the presence of DSM-5 OUD, active suicidality and/or psychosis, and interest in initiating buprenorphine through a shared decision-making process. A Demographic-Treatment History Questionnaire will assess gender, age, race/ethnicity, education, occupation, income, substance use treatment history, and access to a cell phone with text message capability (see Table 2).

**Table 2:** Overview of timing of measurement

Measure	Intake	1- Week	4- Weeks	8- Weeks	12- Weeks
Demographics–treatment history	X				
Distress tolerance scale	X	X	X	X	X
Mirror-tracing persistence task	X	X	X	X	X
Breath-holding task	X	X	X	X	X
Difficulties with emotional regulation scale	X	X	X	X	X
UPPS-P impulsive behavior scale	X	X	X	X	X
Positive affect negative affect schedule	X	X	X	X	X
Decisional balance scale	X				
Timeline follow-Back	X	X	X	X	X
Urine toxicology screens	X	X	X	X	X
Readiness ruler	X	X	X	X	X
Client satisfaction questionnaire	X			X	
System usability scale				X	
Relative subjective count				X	

### Primary outcome

The primary outcome of this study is percentage of days positive for drug use in between assessments (self-report and biochemically verified; see Table 2). The interviewer-delivered Timeline Follow-Back will assess daily opioid use, other illicit substances (e.g., cocaine), and alcohol use. It has demonstrated good reliability [37] and has been validated for the assessment of opioids and other substance use [38]. Urine Toxicology Screens will be administered at all time points to screen for the presence of opioids and other illicit substances.

### Secondary outcomes

This study will also evaluate other clinically-relevant outcomes, including: percentage of days positive for buprenorphine administration in between assessments (self-report via Timeline Follow-Back and biochemically verified via Urine Toxicology Screens) and retention in treatment (see Table 2). Retention will be determined by the total number of clinic visits attended, and treatment discontinuation based on electronic medical record review. This study also seeks to identify potential mechanisms that may underlie the efficacy of iCOPE. Therefore, we have selected several other secondary outcomes of interest. These include motivation, distress tolerance, emotional and behavioral self-regulation skills, and negative affect (see Table 2). The readiness ruler assesses current level of motivation to change opioid use [39]. Self-reported DT will be assessed by the Distress tolerance scale [40], which evaluates general tolerance of psychological distress. Behavioral assessments of DT will include the computerized mirror-tracing persistence task [41] to assess psychological DT and a breath-holding task to assess physical DT. The positive affect and negative affect schedule will be used to evaluate negative affect throughout the study [42]. The difficulties with emotion regulation scale [43] will be used to assess the degree to which respondents experience dysregulated emotional states (emotional self-regulation). The UPPS-S impulsive behavior scale will assess a 5-factor model of impulsivity (behavioral self-regulation) [44].

### Feasibility and acceptability

Treatment satisfaction will be assessed by the client satisfaction questionnaire-8 [45] following the computerized intervention delivered at intake and following the text message portion of the study. Usability and acceptability of the text message intervention will be evaluated by the system usability scale [30–32] while satisfaction will be assessed through *the* relative subjective count (rsc). Low RSC correlates with increased patient satisfaction; high RSC reflects poor acceptability of a technology [33] see Table 2.

## Data analysis

### Overview

Considering this is a pilot study, analyses will have the goal of establishing feasibility, acceptability, and estimation of effect sizes, with modest expectations for rejection of null hypotheses. As a first step, the equivalence of treatment conditions regarding key baseline variables will be assessed. This will involve comparisons of treatment conditions on demographics and baseline levels of potential treatment moderators (e.g., gender and age). Should conditions differ on any characteristic, these variables will be placed in models as interactions with group assignment, along with its main effect, and also in a distinct model with the interaction removed. The model with the lowest Akaike Information Criterion (AIC) will be retained. Other preliminary analyses will include patterns of missing data, dropout rates, distributional properties of dependent and other measures, and correlations among outcome measures. Following the intent-to-treat principle, all randomized participants will be included in the analyses. Given the developmental nature of this study, our primary goal is to yield a stable estimate of the effect size rather than to find statistically significant differences. We are aware of the dangers of relying exclusively on small pilot studies to gauge the promise of novel interventions [46]. These effect size estimates have a large standard error, and we primarily will be examining a pattern of results that is supportive of iCOPE, at which point a full-scale trial will be designed to test for a clinically meaningful effect size. Group means on continuous variables typically begin to stabilize around 15 participants per group. For dichotomous or categorical variables, a larger sample size may be needed to provide stable odds ratios for effect size estimates. Our proposed sample size of 60 participants in iCOPE and 20 participants in HE will allow us to evaluate the potential of iCOPE to improve treatment outcomes while maximizing the number of participants in the iCOPE condition to fully assess the feasibility and acceptability of the intervention. A formal power analysis was not completed as this is a pilot trial, which is not designed to detect statistically significant differences between intervention groups [55].

### Primary pilot trial analyses

Tests of the effects of treatment on the primary outcome variables (abstinence, adherence, retention) will be conducted using a fractional logit model [47] estimated by Generalized Estimating Equations (GEE) [48, 49]. GEE is a quasi-likelihood estimation method of repeated measures for modeling of covariance structures when outcomes are correlated across time. It allows for inclusion of both categorical and continuous independent variables. The fractional logit model can be used for any fractional outcome with a range of 0 to 1 and can be readily implemented using SAS PROC GLIMMIX. An advantage of GEE over ANOVA is that GEE models address nesting by adjusting the standard errors of the test statistics based on the covariances (and variances) of nested observations, rather than depending on calculating differences. These variances and covariances can be modeled based on all data available. The primary, between groups, independent variable in the above GEE is treatment group. Variables measured at baseline will be examined using screening runs prior to primary analyses to see which of these baseline variables are most strongly associated with the outcomes. Those that show significant relations with outcome will be entered as additional covariates in the primary analyses unless there are concerns of multicollinearity. The linear effect of time also will be included as a covariate in these analyses. We will test for non-linear (i.e., linear plus quadratic) effects of time for the repeated measures. Analyses will be conducted separately on two overlapping samples. Following the intent-to-treat principle, all randomized participants will be included in the first set of analyses. This is the most conservative approach and represents our main outcome analysis. We also will conduct analyses on subjects who completed the computer-based intervention, the “as-treated” analysis. Although subject to more bias, especially if dropout is a problem, this latter approach answers more directly the question of intervention efficacy by providing an estimate of the maximal effects attained by an intervention. Similar results with both approaches increases confidence in the findings.

### Exploratory analyses

Potential treatment mechanisms (motivation, distress tolerance, emotional and behavioral self-regulation skills, and negative affect) will be examined separately, rather than simultaneously, given our limited sample size and ability to tease apart the relative contributions of related variables. Mediation analyses will be temporally ordered, such that mediators are assessed after the intervention (controlling for baseline levels of mediators), and outcomes will be assessed at a later time point than the mediators (controlling for baseline levels of outcomes). Though power will be limited, these analyses will at least inform us whether the hypothesized mediators are (a) likely to be influenced by treatment and (b) are likely to associate with our primary outcomes. Conducting these analyses will also provide information to plan a future, larger-scale trial.

## Discussion

The prevalence of OUD has reached epidemic rates in United States, and opioid-involved overdoses are now the country’s leading cause of injury deaths [50]. MOUD, including the use of buprenorphine, is effective at producing significant reductions in illicit opioid use as well as improvements in health-related outcomes (e.g., reduced risk of HIV) [6, 51]. However, despite the many advantages associated with buprenorphine treatment, nearly half of participants are unable to achieve stabilization [7], and many patients lapse or discontinue treatment in the initial weeks [8]. This is notable as early lapses are related to poor treatment prognosis and retention [8, 52]. Given high rates of noncompliance and/or discontinuation, there have been recent calls to find innovative interventions to enhance motivation, adherence, and retention in buprenorphine treatment [53].

Consistent with a negative reinforcement model of substance use, aversive internal states, that occur in the context of early periods of abstinence, may contribute to difficulties with buprenorphine stabilization [54]. Accordingly, an intervention that (a) cultivates motivation for abstinence over and above the reinforcing effects of opioids and (b) teaches adaptive strategies for tolerating physical and psychological discomfort may optimize stabilization on buprenorphine to improve the likelihood of long-term recovery. This Stage 1 behavioral treatment development trial seeks to develop, and pilot test, an innovative digital health intervention that aims to enhance motivation to engage in treatment and increase tolerance of distress to facilitate buprenorphine stabilization by providing skills training and motivational reminders in ‘real-time.’

At the conclusion of this study, our goals are to (1) evaluate the feasibility, acceptability, and preliminary efficacy of the iCOPE intervention components and (2) explore potential mechanisms underlying the effects of the intervention. Results of this study will be used to determine whether to proceed with further testing through a large-scale, fully-powered trial. Even if this study produces null effects of the proposed intervention, the data will still yield valuable information to inform future intervention development efforts.

This work has the potential to improve treatment outcomes by reducing illicit opioid use, increasing adherence/retention, and preventing future incidence of overdose. If successful, this study has high clinical and public health significance by developing and pilot testing the preliminary efficacy of an intervention that may reach a high-risk and vulnerable segment of the population. Further, by leveraging digital health platforms, this proposal has the potential for high scalability and impact.

## Data Availability

Not applicable.

## Abbreviations

OUD: Opioid use disorder
MOUD: Medications for opioid use disorder
DT: Distress tolerance
PFI: Personalized feedback intervention
TAU: Treatment-as-usual
DSM-5: Diagnostic and statistical manual of mental disorders, 5th Edition
RSC: Relative subjective count
AIC: Akaike information criterion
GEE: Generalized estimating equations
